# Involving young people in research investigating comorbidity associated with childhood-onset rheumatic disease: perspectives of a series of focus groups

**DOI:** 10.1186/s41927-025-00492-0

**Published:** 2025-04-09

**Authors:** Sab Siddiq, Jenny Sammy Ainsworth, Clare E. Pain, Eve M.D. Smith, Sizheng Steven Zhao, David M. Hughes, Liza J. McCann

**Affiliations:** 1 Department of Health Data Science, Institute of Population Health, University of Liverpool, Waterhouse Building, Block F, 1-5 Brownlow Street, Liverpool, L69 3GL UK; 2 Institute of Life Course and Medical Sciences, University of Liverpool, Liverpool, UK; 3 Department of Paediatric Rheumatology, Institute in the Park, Alder Hey Children’s NHS Foundation Trust, Liverpool, UK; 4 Department of Women and Children’s Health, Institute of Life Course and Medical Sciences, University of Liverpool, Liverpool, UK; 5 Department of Paediatric Rheumatology, Institute in the Park, Alder Hey Children’s NHS Foundation Trust, Liverpool, UK; 6 Centre for Musculoskeletal Research, Faculty of Biology, Medicine and Health, School of Biological Sciences, Division of Musculoskeletal & Dermatological Sciences, University of Manchester, Oxford Road, Manchester, M13 9PL UK

**Keywords:** Patient and public involvement, Focus groups, Juvenile idiopathic arthritis – Juvenile-onset lupus, Juvenile dermatomyositis, Comorbidity, Childhood-onset

## Abstract

**Background:**

Childhood-onset rheumatic diseases, such as juvenile idiopathic arthritis, juvenile-onset lupus and juvenile dermatomyositis, appear to be associated with an increased risk of comorbidities in adulthood compared to the general population. For the first stage of a research project evaluating this topic, we wanted to capture views from young people with juvenile-onset rheumatic disease to ensure that further work was relevant to their lived experience and priorities. This study aimed to determine (i) which comorbidities young people identify as important, (ii) how they access information about their disease, including comorbidity risk, whether (iii) they would like to hear about the risk of comorbidities whilst they are under paediatric care, and (iv) would be motivated to make lifestyle choices to decrease the risk of potential comorbidities.

**Methods:**

A topic guide based on the proposed study aims was developed, and PowerPoint slides were prepared to facilitate three focus group discussions to gain insights from young people. Focus groups were conducted via video platform, and the views of young people were assimilated using notetaking and an online interactive polling tool.

**Results:**

A total of 18 young people between 10 and 27 years of age participated in the focus groups. Mental health (including depression and anxiety) was described as important comorbidity by 17/18 (94%), followed by obesity or being overweight by 9/18 (50%), heart disease by 7/18 (39%) and stroke by 5/18 (28%) of participants. Young people reported searching United Kingdom National Health Service websites, charity resources, and Google for information on their disease and associated comorbidities. They stated that they would be willing to change their lifestyle to reduce the risk of comorbidities if information were given to them sensitively with clear practical steps for reducing risk.

**Conclusion:**

Three groups of young people identified risk of mental health issues, obesity, and cardiovascular morbidities as particularly important to them. They reported searching online platforms related to their disease and increasingly accessed online resources as they transitioned from paediatric to adult care. Participants thought it would be helpful to provide information on young people’s disease and associated comorbidity in a motivational and sensitive way.

**Clinical trial number:**

Not applicable.

**Supplementary Information:**

The online version contains supplementary material available at 10.1186/s41927-025-00492-0.

## Background

Patient and public involvement (PPI) is an essential part of the research process and needs to be carefully and strategically planned with the assistance of a PPI specialist. It can help to improve research outcomes, contribute to carers’, clinicians’, and policymakers’ decision-making, and facilitate the allocation of provisions to meet patient priorities [1, 2]. United Kingdom (UK) health research funders have recognised the value of patient involvement in research through initiatives such as the National Institute for Health Research PPI strategic plan [3].

Patients with childhood onset rheumatic disease, such as Juvenile Idiopathic Arthritis (JIA), Juvenile-onset Systemic Lupus Erythematosus (jSLE, also known as Childhood-onset SLE), and Juvenile Dermatomyositis (JDM), may be at risk of comorbidities and adverse long-term outcomes later in life [4–6]. This may increase the risk of death or disability and has implications on their quality of life and healthcare utilisation [5, 7]. Studies reporting health outcomes and treatment priorities from the perspective of clinicians or adult patients with multimorbidity demonstrate that the priorities of patients and healthcare professionals do not always align, as patient priorities are primarily based on illness experiences and those of professionals on managing long-term risks of conditions [8]. A study on patient engagement in healthcare research has highlighted that understanding the lived experience of patients receiving medical care may be valuable in framing organisational policy and system interventions [9]. Furthermore, a study to improve comorbidity management in young patients with jSLE highlighted that young people should be involved and their voices be heard to align patient and healthcare professional expectations, promoting better healthcare outcomes [10].

This study aims to determine (i) which comorbidities young people think are important, (ii) how they access information about their disease, including long-term outcomes and risk of comorbidities, whether (iii) young people would like to hear more about the risk of comorbidities whilst they are under paediatric care, and (iv) would be motivated to make lifestyle choices to decrease the risk of potential comorbidities.

## Methods

In a simple study design, young people were invited to participate in focus group discussions utilising the infra-structure of established organisations. A sample size of three focus groups was chosen, partly for pragmatic reasons, but also recognising from previous work that 2–3 focus groups have been found to identify at least 80% of themes on a topic [11]. Although a discussion framework was utilised (shown in supplementary material), open discussion and free text comments were allowed and captured as described below. Notes were taking during meetings by 3 authors (SS, JSA, LJM) and responses from the on-line voting tool used (Poll Everywhere) were recorded. Themes were identified from the transcripts with the help of our Young Persons and Family Coordinator (JSA).

### Participants

Following ethics approval, young people were invited to three focus groups, coordinated with the help of established PPI organisations that were experienced in research involving young people and relevant to the diseases evaluated. Authors (SS and LJM) co-facilitated focus groups with PPI Leads of each group. The groups participating in focus group discussions were (i) Generation R Liverpool, a network alliance of young people advisory groups based around the UK that aims to support the design and delivery of paediatric health research (lived experience of healthcare but not specifically of rheumatic diseases) [12], (ii) Your Rheum, a group of 11–24 years old with childhood onset rheumatic disease that offers advice to researchers [13, 14]; and (iii) Lupus UK, a young people group with Lupus [15].

### Preparation for focus group discussions

The authors formulated a topic guide, including questions to be asked within focus groups (shown in supplementary material) that covered the study aims. A set of PowerPoint slides was prepared based on questions and reviewed by the PPI specialist to ensure that they were age appropriate. The language was adjusted to ensure the presentation was as simple and engaging as possible. Before each meeting, slides were reviewed by the PPI leads of the respective groups. Based on their feedback on content and language, further adaptations were made. An online polling tool, Poll Everywhere, was embedded within the PowerPoint presentation to allow participants to interactively engage in discussion and elicit real-time opinions during the presentation [16]. Prior to conducting the focus groups, the presentation was piloted among clinical members of our research team to ensure that the voting system worked well and that the content and language were appropriate for patient groups.

### Focus groups

Focus groups were conducted via video platform. Each session started with a welcome and introductions followed by ice-breaker questions. After an overview of the project aims, we described the three diseases (JIA, jSLE, and JDM) we plan to evaluate in future work on comorbidities. To achieve aim 1 of the study (to assess which comorbidities were important to participants), examples of comorbidities found in adult-onset disease in published literature were presented to the groups [17–22]. Young people were asked to consider these comorbidities and based on their experience with their condition, reflect on which comorbidities were important to them. To achieve aim 2 (understanding how young people access information relevant to their conditions), the groups were shown the results of ‘Answer The Public’, a Google search query, as an example of a way that the public may access information online relating to the diseases being studied [23]. This was used as an introduction to promote discussion on how young people may access information about their condition. To achieve aim 3 and 4 (whether young people would like to hear more about the risk of comorbidities whilst they are under paediatric care and if they would be motivated to make lifestyle choices to decrease the risk of potential comorbidities), they were asked open-ended questions to elicit opinions.

## Results

Eighteen young people, 11 females and 7 males, participated in focus groups. They were between 10 and 27 years old and attended events from 12 regions/cities across the UK. The demographic details of participants are shown in Table 1.

**Table 1:** Basic demographics of focus group participants

Group Name (meeting date)	Number of participants	Mean age in years (range)	Proportion of females
**Generation R** (22/01/2022)	9	Mean 15.5 (10–21)	44%
**Your Rheum** (27/01/2022)	7	Mean 17.5 (11–24)	71%
**Lupus UK** (12/05/2022)	2	Mean 24.5 (22–27)	100%

### Aim 1: Determining which comorbidities are important to young people

Young people were asked to reflect on the listed comorbidities taken from published literature, including heart disease, high blood pressure, obesity or being overweight, bone disease (osteoporosis or osteopenia), stroke, diabetes, mental health disorders, and liver disease, and asked to state what they thought was important to them. Young people were also able to include other comorbidities which may be important to them. Most participants (17/18; 94%) consistently highlighted mental health as important (Table 2). Other comorbidities rated highly were obesity or overweight (9/18; 50%), heart disease (7/18; 39%), and stroke (5/18; 28%). In addition, participants mentioned liver disease (2/18, 11%), diabetes (2/18, 11%), and infertility (2/18, 11%). Some participants opted for ‘other’ and indicated that uveitis and lung disease were important.

**Table 2:** Results of focus group discussion identifying which comorbidities are important to young people

Comorbidity	Number of participants identifying each comorbidity as important to them in focus groups (total participants)			Total number of Young People (%) choosing each comorbidity as important across the groups
	Generation *R* (9)	Your Rheum (7)	Lupus UK (2)	
Mental health/Depression/ Anxiety	9	7	1	**17 (94)**
Overweight/Obesity	6	3	0	**9 (50)**
Heart disease/Cardiovascular risk	0	7	0	**7 (39)**
Stroke	0	5	0	**5 (28)**
Diabetes	2	0	0	**2 (11)**
Liver disease	0	1	1	**2 (11)**
Infertility	0	1	1	**2 (11)**
Infection/Covid	1	0	0	**1 (6)**
Hypertension	0	0	1	**1 (6)**
Uveitis	0	1	0	**1 (6)**
Lung involvement	0	1	0	**1 (6)**

### Aim 2: Determining how young people access information about their disease

Young people in these groups gathered information regarding their health and illness in various ways. Eight out of nine (89%) participants from the Generation R group said they used Google to search online for information. Other sources of information included the use of UK National Health Service (NHS) websites (3/9; 33%), National Institute for Clinical Excellence, NICE (1/9; 11%), Internet Explorer (1/9; 11%), YouTube (1/9; 11%) and by asking their parents (2/9; 22%). One out of nine (11%) participants chose “videos from people with the condition” but did not specify a specific source or social media platform for videos. They expressed concern about the reliability of sources of online information and the need to be aware of misinformation. Participants stated that the source of information they used depended on the questions they were asking. When searching for information about health and disease, they ensured that the source of information was reliable by cross-checking with official NHS websites. However, they were happy to use Google if they were looking for information related to school and homework.

Similarly, the Your Rheum group (*n* = 7) described using a mixture of sources of information. They relied on rheumatologists or other healthcare professionals for more serious matters. Two out of seven (29%) participants looked at NHS websites and mentioned that their preferred sources of information had changed over the years. When under paediatric care, they relied on (and trusted) information from their rheumatologist or parents. Participants expressed that under paediatric care, they felt more supported, but as they transitioned to adult services, they felt that things appeared more rushed, and they were expected to do more themselves. At this stage, they were more inclined to access information from social media or Arthur’s Place, an online magazine that provides resources for young people with arthritis [24]. One out of seven (14%) participants mentioned using Arthur’s Place for emotional issues. They also used the Versus Arthritis website (2/7; 29%), the National Rheumatoid Arthritis Society, NRAS (2/7; 29%), Health Unlocked (a social networking service for health), Instagram, online support groups and other online forums.

Participants of the Lupus UK group (*n* = 2) described searching for disease-related information on Google but validating this information with other relevant websites, such as NHS resources. The Lupus UK website and its associated online platforms were considered a reliable source of information and support. The Health Unlocked platform moderated by Lupus UK and a WhatsApp group facilitated by Lupus UK were highlighted as helpful. Speaking to medical professionals was always preferable to online sources and significant to young people.

### Aim 3: Determining whether young people would like to hear more about the risk of comorbidities while under paediatric care

This section focused on how much young people would like to know about their disease and the potential associated risk of comorbidities. Young people shared that their opinions changed over time. At an earlier stage of their condition or when they were adolescents, they did not want to know details. However, they expressed that they would like to learn more about their illness as they progressed through adolescence and into adulthood. A key point from participants was that information should be given sensitively and at the right time. They also thought that young people needed to be given practical support to know how to deal with it.

When participants reflected on the long-term impact of living with these diseases, they had some concerns about the impact of disease, e.g. “struggling at school” and “limited access to sports” in the short term; “pregnancy outcomes”, “affecting future”, “affecting work-life”, “impact on quality of life” in medium-term; and “earlier death due to complications” in long-term (detailed in supplementary material). They also raised concerns about the emotional impact of disease, describing feelings such as “loneliness”, “isolation”, “sad” and “overwhelming”. However, reflecting on the long-term effects of the disease led to considering positive life changes, including opting to “manage lifestyle, diet, exercise” and “live life as normally as possible.”

Specific themes emerged based on participant responses on whether knowing more about long-term outcomes would be helpful or anxiety-inducing. Many participants thought that more information from professional sources would help them. One participant mentioned, “Yes, because I could prepare myself for the potential long-term outcomes, so it is less of a shock if it does happen.” Similarly, another related ‘’I would want to know ways to help it and ways that will make it worse for me, and it is important to know that you can still lead a normal life.’’ Also, a respondent emphasised that the availability of information could motivate them to make life changes. Others agreed that it could be useful as it could help prepare for the future, but recognised the uncertainty of whether they would be affected by a comorbidity. One young person stated, “It would help me to know and be prepared. It may cause more anxiety, but I would want to know for similar reasons.”

Some participants expressed that more information about long-term outcomes related to their disease could be scary. One respondent suggested it may be “more of a burden.” Another insightfully raised the possibility that “stress may make young people more likely to smoke or promote risk-taking behaviour/anxiety.’’ A participant expressed that ‘’more information about outlook or comorbidity may be confusing for young people, increasing anxiety, and anxiety may affect education and cause difficulty concentrating.” Similarly, another participant mentioned that ‘’because my disease is a new diagnosis, I overthink information, and particularly long-term effects would be too much and cause anxiety.’’ A participant resonated that it would be ‘’helpful but also scary and could cause people to obsess about serious conditions.’’ Furthermore, they emphasised that information on the long-term outcome or comorbidities should not be given at a younger age, or first diagnosed.

Participants highlighted that the benefit of the availability of information “depends on the person, age and the disease.” One participant described that “it depends on the treatment of the disease and how people around you deal with it; if a person accepts that this is how it is, knowing about long-term outcomes probably would not change anything. However, if it makes you more aware, you may be able to implement steps to help.’’ Some respondents suggested that healthcare professionals need to weigh up benefits and risks. They thought that if there was a high probability of getting a disease or comorbidity, it may be important to tell a young person. They also stated that knowledge of risk may impact mental health. They thought it was important to be positive where possible, e.g. ‘’low chance of cancer, but if this happens, we have treatment’’. They thought positivity was reassuring. Other participants suggested that it was important for healthcare professionals to ‘’read the signs of how much information young people want.’’.

### Aim 4: Determining if young people would be motivated to make lifestyle choices to decrease the risk of potential comorbidities

Considering if knowing about the risk of comorbidities would help to manage lifestyle choices in different ways and decrease these risks, participants were optimistic in their response. They thought more information would enable them to manage their disease, lifestyles, and choices productively. One emphasised that ‘’if I knew the outcomes, I would try to change my lifestyle to try and limit the effects as much as possible.’’ Similarly, another saw this opportunity as having ‘’a significant impact on life as your doctors and care team are so knowledgeable that if they give you advice, you will want to follow it for the good of your health.’’ ‘’It is an opportunity to be more cautious.’’ Another participant responded, “Yes, I want to help it improve, not make it worse, and it would reduce risk factors.” Others suggested that they would exercise more, follow doctors’ advice, want to live as normally as possible for young people, and manage lifestyle, diet, exercise and pacing. Some participants used weight as an example. They described this as difficult to control but were ready to accept and change lifestyles. One participant commented that they now (as a young adult) understand that weight can impact joints. However, they suggested not putting too much pressure on young people but giving balanced information and being sensitive. One participant stated it “depends - you would be aware, but then you have to think about implementation, ensuring it’s practical, local and realistic”, emphasising the need to practically support young people to make the necessary changes through integrated, personalised interventions to reduce the risks of comorbidities. They articulated that it can be challenging for young people when they do not have complete control of their disease, but if a professional says this will help, young people will follow it. A theme of empowerment emerged as young people saw the importance of letting a person know how to make changes (such as losing weight) in a way that was practical or in a stepwise fashion. Health Coaching was mentioned - used by GP practices, where health coaches work with people to give them small steps to change, e.g. jumping a few times whilst waiting for the kettle to boil if making a cup of tea. Other participants thought that using tools like Health Coaching could help but would be age dependent.

## Discussion

To the best of our knowledge, no previous studies have asked young people with juvenile onset rheumatic disease what comorbidities are important to them. In this study, young people with juvenile-onset rheumatic disease participating in Your Rheum and Lupus UK focus groups, as well as those from GenerationR with an understanding of chronic disease, identified mental health, being obese or overweight, heart disease and stroke as important comorbidities, followed by liver disease, diabetes, and fertility-related complications of their disease. They also emphasised how chronic diseases and medications used in treatment regimens may exacerbate the risk of obesity, mental illness, infections, and infertility, among other comorbidities. The insights gained from young people during these PPI groups will be instrumental in informing further research evaluating comorbidities relating to JIA, jSLE and JDM.

The impact on mental health was a key concern for young people in this study, which aligns with previous literature. A North American study has identified that clinician-diagnosed or self-diagnosed mental health disorders were prevalent in young people with JIA, jSLE and JDM, with the most commonly reported problems being anxiety (81/123, 66%), depression (65/123, 53%) or adjustment disorder (45/123, 37%) [25]. Barriers to accessing mental health services included concerns that mental health professionals would not understand the rheumatological disease. A high proportion of patients in this study (50/85, 60%) reported using online mental health resources. There is evidence to suggest that the risk of mental health disorders is higher in childhood onset rheumatic disease than in those with adult-onset disease, raising the importance of identification, education and early intervention [26].

The participants of this study sought information on their condition, treatment and management using different sources, including NHS websites and resources from charities related to their disease. They suggested that when they searched for information using search engines, such as Google, they cross-checked the accuracy of information with other service provider information, such as NHS websites or charitable organisations. This highlighted the importance of considering a variety of online platforms to disseminate disease related information and provide educational material for patient groups. An Australian study investigated how young adults (*n* = 165, aged 18–24 years) interact with different social media platforms for health and health information using a web-based conversation methodology. They found that 112/165 (67.8%) of participants used social media to talk or learn about health. In particular, Facebook newsfeeds often resulted in seeing health-related content. YouTube was considered helpful, with the video format easy to learn from and credibility judged instinctively or by cross-checking multiple videos. Instagram was a source of inspiration for wellness, healthy lifestyle and fitness. A smaller group used Pinterest for health reasons, especially for recipes or workouts. Twitter, Tumblr and Snapchat were rarely used for health information [27].

A study on whether social media, along with web-based education, led to improved medication adherence among adolescents and young adults with SLE showed that an online educational intervention led to an improvement in medication adherence for all participants, but adherence was further enhanced for participants engaging in disease-centred social media forums with SLE peers compared to those without social media access [28]. A review evaluating the effects of social media on paediatric rheumatic disease suggested that social media facilitated access to online communities for patients and their families [29]. Adolescents and young adults with rare rheumatic disease have the potential to benefit significantly from online interactions with peers who share common experiences. Social media may also allow family communities to collaborate regardless of geographic distance, support each other, and advocate for their children to improve the care and awareness of the disease [29]. A study on a self-management smartphone app system for young people with JIA demonstrated that using the app provided them with opportunities and motivation for better self-management via medication reminders, easy access to information, advice, support, understanding of their behaviours, and control of their disease activity. Reading other patients’ success stories and reflecting on their own disease management increased their confidence to cope with future challenges [30].

A study on podcasting as an innovative tool to enhance osteoarthritis research dissemination and education suggested that podcasting as a source of educational content may also be applicable to paediatric services [31]. Podcasts could allow young people to learn more about their disease and enable discussions. A wide range of patients may be reached by linking research outcomes to various social media platforms, enhancing knowledge of their medical condition. Social media may facilitate a platform for continued discussion among all stakeholders involved, including healthcare experts, patients, their carers and families [32].

Responses to would knowing more about long-term outcomes or co-morbidities hinders or helps to manage lifestyle, suggested that the young people included in this study would be willing to change their lifestyle to decrease the risk of comorbidities provided that information was given to them later in the course of their illness, when they were at an older age, and presented sensitively, including simple steps for how they can practically work on decreasing their risk. It is important to note that young people who choose to participate in focus groups may be particularly motivated, and this may not reflect the thoughts and feelings of all young people with childhood onset rheumatic disease. If questions are directed towards young people without any health issues or involvement in any health or research related groups, different results may be demonstrated. Additionally, more work is needed to determine if lifestyle changes will reduce long-term risk of comorbidity. Accepting these limitations, it may be appropriate to educate about the risks of comorbidities whilst preparing young people to transition to adult services. The European Alliance of Associations for Rheumatology standards and recommendations for transitional care of young people with rheumatic disease advised that holistic care includes the need to be future focused and not limited to young adulthood to ensure optimal well-being [33]. Young people may be receptive at this time to a multi-disciplinary approach that includes recommended lifestyle changes (where appropriate) and health choices that minimise the risk of comorbidities later in life.

A qualitative study involving children and young people with JDM with a range of disease duration, severity and experiences highlighted themes of “feeling different”, “sick”, “steroidal and scared”, and feeling “uncertainty” depending on what else was happening due to JDM itself or changing personal and social circumstances [34]. However, as far as we are aware, previous studies have not specifically evaluated the perceptions of young people with juvenile-onset rheumatic disease about comorbidities and counselling of risk factors. This study brings together some important points for clinicians to consider whilst supporting young people’s journey to adulthood. By aligning the clinician perspective with patient priorities, better patient outcomes could be ensured.

There are a number of limitations to this study. A major limitation is that each group, particularly the Lupus UK group, had small numbers of participants. The unbalanced number of participants in each group did not allow for robust comparison between groups and the total number of participants (*n* = 18) in this study was modest. However, being in small groups, participants were able to engage in detailed discussions and made equal contributions to generate effective ideas.

A sample size of 3 focus groups was chosen, but we acknowledge, particularly in view of the small number of patients in the jSLE focus group, that a higher number of focus groups may have provided additional value. We recognise that the small number of participants may not have captured overall concerns about many comorbidities relevant to rheumatic disease such as growth related issues, joint deformities, intellectual capability, renal dysfunction and others. We also acknowledge that GenerationR is not a group specific to rheumatic conditions, but young people in this group are experienced in the design and delivery of paediatric health research and have an appreciation of chronic disease. Themes identified from this group were in line with those of the 2 rheumatic-specific focus groups.

Due to social or language restrictions, patients who choose to join PPI groups might not represent the whole disease population, and tend to be more motivated patients who are actively involved in their care and receptive to health promotion activities. We did not evaluate ethnicity, sexual orientation, or social factors within this study. Because we used Poll Everywhere as a tool to gather responses, we were unable to determine the age of participants responding in any particular way and therefore we could not determine if age related factors or developmental stage influenced opinions. It would have been interesting to know if age or developmental stage influenced the responses given.

This study relied on self-reported experiences and opinions rather than objective clinical data, which we accept can introduce recall bias. We did not use a standardized framework to quantify importance of comorbidities as we wanted young people to simply judge what was important to them without influence. We acknowledge that participant responses may be influenced by recent experiences rather than accurate representation of their overall disease course. Furthermore, in view of the small sample size, we did not explore individual patient characteristics that could have influenced patient perspectives on comorbidities such as disease severity, disease duration, treatment type or education received. We recognise that risk of comorbidity may vary according to disease sub-type or disease severity. A large German database study found that children with systemic or enthesitis-related arthritis were more likely to be obese than other JIA subtypes and higher corticosteroid dose, disease activity and functional limitations influenced risk of being overweight [35]. Although there is conflicting evidence, other studies have supported a correlation between both functional impairment and disease activity with obesity and also increased cardiovascular risk factors in obese JIA patients [36]. Cardiovascular risks in patients with jSLE and JDM are multi-factorial but obesity can be contributory and have a negative impact of health related quality of life [36].

Future research involving PPI of young people with juvenile rheumatic disease is recommended to identify specific challenges and opportunities related to the disease and associated comorbidities, including minority groups. It would also be interesting to evaluate what role social media and forums such as TikTok have in young peoples’ decision making process. Finally, it would be helpful to utilise the qualitative findings from this study in a survey based approach to determine if findings are generalizable to a larger population of children and young people with rheumatic disease.

## Conclusions

This study showed that mental health, obesity, and cardiovascular risk were key comorbidities for young people who participated in these focus groups. This study demonstrated that young people would be receptive to education about the risk of comorbidities and would be willing to make lifestyle choices to minimise risks if the information was given to them at the right stage in their illness and in a sensitive, practical way. An opportunistic time may be when young people are being prepared to transition to adult services, supported by multidisciplinary teams and disease-specific online resources. Young people’s opinions elicited in this work will be used to inform further research evaluating comorbidities relating to JIA, jSLE and JDM.

## Electronic supplementary material

Below is the link to the electronic supplementary material.

Supplementary Material 1

## Data Availability

All data generated or analysed during this study are included in this published article and its supplementary information files.

## Abbreviations

JDM: Juvenile Dermatomyositis
JIA: Juvenile Idiopathic Arthritis
jSLE: Juvenile-onset systemic Lupus erythematosus
NHS: National Health Service
NICE: National Institute for Clinical Excellence
NRAS: National Rheumatoid Arthritis Society
PPI: Patient and Public Involvement and Engagement
SLE: Systemic Lupus Erythematosus
UK: United Kingdom
US: United States
