# Supplementary material for: Involving young people in research investigating comorbidity associated with childhood-onset rheumatic disease: perspectives of a series of focus groups
Source: BMC Rheumatol. 2025 Apr 9;9:40. doi: 10.1186/s41927-025-00492-0 (PMC11980279; doi:10.1186/s41927-025-00492-0)
Supplement: Supplementary file 1 — Supplementary Material 1 [file 41927_2025_492_MOESM1_ESM.doc]

**Supplementary material**

**Supplementary material 1:**

**Topic guide questions were used during each focus group alongside the presentation.**

1. How are you feeling today? [Icebreaker/practice of using voting tool].
2. Where are you joining us from? [Icebreaker/practice of using voting tool].
3. What is the best flavour of your ice cream? [Icebreaker/practice using voting tool].
4. What words come to mind when you think of comorbidity?
5. What words come to mind when you think of long-term outcomes?
6. Do you use Google search?
7. What other sources do you use?
8. Which sources of information do you find most useful?
9. Which comorbidity is important to you?
10. Would knowing more about the long-term outcome of the disease help or cause more anxiety?
11. Would it make you manage your lifestyle / your disease/ choices differently? How?
12. Would you be happy for us to return later to discuss the study results and get your views?
13. Anything you particularly enjoyed about the event?
14. How could we have improved the event?

**Supplementary material 2:**

**Young people’s responses while reflecting on comorbidity and long-term outcomes of living with rheumatic disease.**

| **Focus groups’ responses** | | |
| --- | --- | --- |
| **Generation R** | **Your Rheum** | **LUPUS UK** |
| Unfortunate. | Uncertainty. | Not knowing. |
| Can sometimes increase the risk for poorer health. | Lack of understanding. | Impact of diet. |
| Complex. | Loneliness. | Prevention of flares. |
| Impact on life. | Additional symptoms or complications. | Relationship concerns / pregnancy outcomes. |
| Affecting work-life. | Hospital appointments. |  |
| Affecting your future. | Relationships affected. |  |
| Limiting. | Impact on quality of life. |  |
| Limited access to sports / missing out / struggling at school. | How you carry on living life with chronic disease. |  |
| Burden / lots to deal with. | More vulnerable / isolation. |  |
| Overwhelming / scary. | High risk. |  |
| Sad. | Dependence on medication. |  |
| Serious. | A worse outcome / earlier death due to complications. |  |
| Disability. | Difficulty. |  |
| Disadvantage. |  |  |
| Need support. |  |  |
| Draining. |  |  |
| Continuing / never ending. |  |  |
| Being quite sick. |  |  |
